# Supplementary material for: Association between Vitamin D Receptor Gene Polymorphisms and Periodontal Bacteria: A Clinical Pilot Study
Source: Biomolecules. 2022 Jun 15;12(6):833. doi: 10.3390/biom12060833 (PMC9221517; doi:10.3390/biom12060833)
Supplement: Supplementary file 1 [file biomolecules-12-00833-s001.zip › TABLE S2.pdf]

Table S2. For each bacterial strain on top distributions of genotype and allele frequencies, down haplotype analysis of SNPs VDR FokI (Rs2228570), VDR BsmI (Rs1577710), VDR ApaI (Rs7975232) and VDR TaqI (Rs731236) in health controls and periodontitis patients .

**Aggregatibacter Actinomycetemcomitans**					
			LOW (N=36)	HIGH (N=14)	P value
**VDR FokI** **rs2228570** Exon2 c.2T>C (*f>F*)* *p.Met1Thr*	Genotypes (%)	C/C (F/F)	13 (36)	1 (7)	**0.034708**
		C/T (F/f)	14 (39)	11 (79)	
		T/T (f/f)	9 (25)	2 (14)	
	Alleles (%)	C (F)	40 (56)	13 (46)	0.41
		T (f)	32 (44)	15 (54)	
	HW (p)		0.2	0.1	
**VDR BsmI** **rs1544410** Intron 8 c.1024+283A>G(*B>b*)*	Genotypes (%)	A/A (B/B)	1 (3)	0 (0)	0.75
		A/G (B/b)	6 (17)	2 (14)	
		G/G (b/b)	29 (81)	12 (86)	
	Alleles (%)	A (B)	8 (11)	2 (7)	0.55
		G (b)	64 (89)	26 (93)	
	HW (p)		0.35	1	
**VDR ApaI** **rs7975232** Intron 8 c.1025-49A>C(*A>c*)*	Genotypes (%)	A/A (A/A)	5 (14)	0 (0)	0.28
		A/C (A/a)	17 (47)	9 (64)	
		C/C (a/a)	14 (39)	5 (36)	
	Alleles (%)	A (A)	27 (38)	9 (32)	0.61
		C (a)	45 (62)	19 (68)	
	HW (p)		1	0.22	
**VDR TaqI** **rs731236** Exon 9 c.1056T>C (*T>t*) p.Ile352=	Genotypes (%)	T/T (T/T)	32 (89)	13 (93)	0.67
		T/C (T/t)	4 (11)	1 (7)	
		C/C (t/t)	0 (0)	0 (0)	
	Alleles (%)	T (T)	68 (94)	27 (96)	0.68
		C (t)	4 (6)	1 (4)	
	HW (p)		1	1	

**Aggregatibacter Actinomycetemcomitans**								
**Haplotype**				**Total frequency**	**Clinical groups frequency**		**p-value**	**OR (95% CI)**
**FokI**	**BsmI**	**ApaI**	**TaqI**		**LOW**	**HIGH**		
T *(f)*	G *(b)*	C *(a)*	T *(T)*	0.287	0.247	0.214	0.66	0.793 (0.277-2.266)
C *(F)*	G *(b)*	C *(a)*	T *(T)*	0.260	0.266	0.392	**0.25**	1.702 (0.677-4.281)
C *(F)*	G *(b)*	A *(A)*	T *(T)*	0.200	0.228	0	**0.005**	-
T *(f)*	G *(b)*	A *(A)*	T *(T)*	0.112	0.104	0.287	**0.029**	3.322 (1.088-10.140)
C *(F)*	A *(B)*	C *(a)*	T *(T)*	0.05	0.04	0.07	0.62	1.568 (0.255-9.648)

Porphyromonas Gingivalis					
			LOW (N=28)	HIGH (N=22)	P value
**VDR FokI** **rs2228570** Exon2 c.2T>C (*f>F*)* *p.Met1Thr*	Genotypes (%)	C/C (F/F)	12 (43)	2 (9)	**0.022173**
		C/T (F/f)	10 (36)	15 (68)	
		T/T (f/f)	6 (21)	5 (23)	
	Alleles (%)	C (F)	34 (61)	19 (43)	0.081
		T (f)	22 (39)	25 (57)	
	HW (p)		0.23	0.19	
**VDR BsmI** **rs1544410** Intron 8 c.1024+283A>G(*B>b*)*	Genotypes (%)	A/A (B/B)	1 (4)	0 (0)	0.087
		A/G (B/b)	7 (25)	1 (5)	
		G/G (b/b)	20 (71)	21 (95)	
	Alleles (%)	A (B)	9 (16)	1 (2)	**0.022462**
		G (b)	47 (84)	43 (98)	
	HW (p)		0.53	1	
**VDR ApaI** **rs7975232** Intron 8 c.1025-49A>C(*A>c*)*	Genotypes (%)	A/A (A/A)	4 (14)	1 (5)	0.22
		A/C (A/a)	16 (57)	10 (45)	
		C/C (a/a)	8 (29)	11 (50)	
	Alleles (%)	A (A)	24 (43)	12 (27)	0.10
		C (a)	32 (57)	32 (73)	
	HW (p)		0.47	1	
**VDR TaqI** **rs731236** Exon 9 c.1056T>C (*T>t*) p.Ile352=	Genotypes (%)	T/T (T/T)	26 (93)	19 (86)	0.44
		T/C (T/t)	2 (7)	3 (14)	
		C/C (t/t)	0 (0)	0 (0)	
	Alleles (%)	T (T)	54 (96)	41 (93)	0.45
		C (t)	2 (4)	3 (7)	
	HW (p)		1	1	

Porphyromonas Gingivalis								
**Haplotype**				**Total frequency**	**Clinical groups frequency**		**p-value**	**OR (95% CI)**
**FokI**	**BsmI**	**ApaI**	**TaqI**		**LOW**	**HIGH**		
T *(f)*	G *(b)*	C *(a)*	T *(T)*	0.286	0.170	0.464	**0.0017**	4.145 (1.658-10.361)
C *(F)*	G *(b)*	C *(a)*	T *(T)*	0.260	0.258	0.207	0.51	0.730 (0.284-1.279)
C *(F)*	G *(b)*	A *(A)*	T *(T)*	0.200	0.239	0.202	0.62	0.788(0.302-2.058)
T *(f)*	G *(b)*	A *(A)*	T *(T)*	0.112	0.154	0.036	**0.04**	0.202(0.035-1.149)
C *(F)*	A *(B)*	C *(a)*	T *(T)*	0.05	0.09	0.02	0.18	0.225 (0.036-1.423)

Porphyromonas Endodontalis					
			LOW (N=26)	HIGH (N=24)	P value
**VDR FokI rs2228570** Exon2 c.2T>C (*f>F*)* *p.Met1Thr*	Genotypes (%)	C/C (F/F)	12 (46)	2 (8)	**0.010462**
		C/T (F/f)	9 (35)	16 (67)	
		T/T (f/f)	5 (19)	6 (25)	
	Alleles (%)	C (F)	33 (63)	20 (42)	**0.029180**
		T (f)	19 (37)	28 (58)	
	HW (p)		0.22	0.11	
**VDR BsmI rs1544410** Intron 8 c.1024+283A>G(*B>b*)*	Genotypes (%)	A/A (B/B)	1 (4)	0 (0)	0.20
		A/G (B/b)	6 (23)	2 (8)	
		G/G (b/b)	19 (73)	22 (92)	
	Alleles (%)	A (B)	8 (15)	2 (4)	0.06
		G (b)	44 (85)	46 (96)	
	HW (p)		0.47	1	
**VDR ApaI rs7975232** Intron 8 c.1025-49A>C(*A>c*)*	Genotypes (%)	A/A (A/A)	4 (15)	1 (4)	0.16
		A/C (A/a)	15 (58)	11 (46)	
		C/C (a/a)	7 (27)	12 (50)	
	Alleles (%)	A (A)	23 (44)	13 (27)	0.07
		C (a)	29 (56)	35 (73)	
	HW (p)		0.69	0.64	
**VDR TaqI rs731236** Exon 9 c.1056T>C (*T>t*) p.Ile352=	Genotypes (%)	T/T (T/T)	24 (92)	21 (88)	0.57
		T/C (T/t)	2 (8)	3 (12)	
		C/C (t/t)	0 (0)	0 (0)	
	Alleles (%)	T (T)	50 (96)	45 (94)	0.58
		C (t)	2 (4)	3 (6)	
	HW (p)		1	1	

Porphyromonas Endodontalis								
**Haplotype**				**Total frequency**	**Clinical groups frequency**		**p-value**	**OR (95% CI)**
**FokI**	**BsmI**	**ApaI**	**TaqI**		**LOW**	**HIGH**		
T *(f)*	G *(b)*	C *(a)*	T *(T)*	0.287	0.133	0.492	**0.00009**	6.427(2.395-17.249)
C *(F)*	G *(b)*	C *(a)*	T *(T)*	0.260	0.290	0.162	0.12	0.464 (0.174-1.236)
C *(F)*	G *(b)*	A *(A)*	T *(T)*	0.200	0.261	0.212	0.53	0.746 (0.294-1.897)
T *(f)*	G *(b)*	A *(A)*	T *(T)*	0.112	0.143	0.02	**0.04**	0.178 (0.028-1.140)
C *(F)*	A *(B)*	C *(a)*	T *(T)*	0.05	0.06	0.04	0.61	0.634 0.(0.104-3.846)

Treponema Denticola					
			LOW (N=22)	HIGH (N=28)	P value
**VDR FokI rs2228570** Exon2 c.2T>C (*f>F*)* *p.Met1Thr*	Genotypes (%)	C/C (F/F)	10 (45)	4 (14)	**0.045747**
		C/T (F/f)	9 (41)	16 (57)	
		T/T (f/f)	3 (14)	8 (29)	
	Alleles (%)	C (F)	29 (66)	24 (43)	**0.021908**
		T (f)	15 (34)	32 (57)	
	HW (p)		0.65	0.47	
**VDR BsmI rs1544410** Intron 8 c.1024+283A>G(*B>b*)*	Genotypes (%)	A/A (B/B)	0 (0)	1 (4)	0.60
		A/G (B/b)	3 (14)	5 (18)	
		G/G (b/b)	19 (86)	22 (79)	
	Alleles (%)	A (B)	3 (7)	7 (12)	0.34
		G (b)	41 (93)	49 (88)	
	HW (p)		1	0.35	
**VDR ApaI rs7975232** Intron 8 c.1025-49A>C(*A>c*)*	Genotypes (%)	A/A (A/A)	1 (4)	4 (18)	0.14
		A/C (A/a)	14 (50)	12 (55)	
		C/C (a/a)	13 (46)	6 (27)	
	Alleles (%)	A (A)	20 (45)	16 (29)	0.08
		C (a)	24 (55)	40 (71)	
	HW (p)		1	0.38	
**VDR TaqI rs731236** Exon 9 c.1056T>C (*T>t*) p.Ile352=	Genotypes (%)	T/T (T/T)	20 (91)	25 (89)	0.84
		T/C (T/t)	2 (9)	3 (11)	
		C/C (t/t)	0 (0)	0 (0)	
	Alleles (%)	T (T)	42 (95)	53 (95)	0.85
		C (t)	2 (5)	3 (5)	
	HW (p)		1	1	

**Treponema Denticola**								
**Haplotype**				**Total frequency**	**Clinical groups frequency**		**p-value**	**OR (95% CI)**
**FokI**	**BsmI**	**ApaI**	**TaqI**		**LOW**	**HIGH**		
T *(f)*	G *(b)*	C *(a)*	T *(T)*	0.287	0.129	0.431	**0.0009**	5.259 (1.865-14.823)
C *(F)*	G *(b)*	C *(a)*	T *(T)*	0.260	0.416	0.165	**0.004**	0.267 (0.105-0.683)
C *(F)*	G *(b)*	A *(A)*	T *(T)*	0.200	0.187	0.168	0.79	0.869 (0.308-2.454)
T *(f)*	G *(b)*	A *(A)*	T *(T)*	0.112	0.176	0.05	**0.05**	0.278 (0.070-14.823)
C *(F)*	A *(B)*	C *(a)*	T *(T)*	0.05	0	0.05	0.17	-

Tannerella Forsythia					
			LOW (N=33)	HIGH (N=17)	P value
**VDR FokI rs2228570** Exon2 c.2T>C (*f>F*)* *p.Met1Thr*	Genotypes (%)	C/C (F/F)	13 (39)	1 (6)	**0.034969**
		C/T (*F/f*)	13 (39)	12 (71)	
		T/T (*f/f*)	7 (21)	4 (24)	
	Alleles (%)	C (*F*)	39 (59)	14 (41)	0.08
		T (*f*)	27 (41)	20 (59)	
	HW (p)		0.29	0.14	
**VDR BsmI rs1544410** Intron 8 c.1024+283A>G(*B>b*)*	Genotypes (%)	A/A (*B/B*)	1 (2)	0 (3)	0.26
		A/G (*B/b*)	7 (16)	1 (21)	
		G/G (*b/b*)	25 (82)	16 (76)	
	Alleles (%)	A (*B*)	9 (14)	1 (3)	0.09
		G (*b*)	57 (86)	33 (97)	
	HW (p)		0.47	1	
**VDR ApaI rs7975232** Intron 8 c.1025-49A>C(*A>c*)*	Genotypes (%)	A/A (*A/A*)	4 (12)	1 (1)	0.41
		A/C (*A/a*)	15 (45)	11 (52)	
		C/C (*a/a*)	14 (42)	5 (38)	
	Alleles (%)	A (*A*)	23 (35)	13 (38)	0.73
		C (*a*)	43 (65)	21 (62)	
	HW (p)		1	0.3	
**VDR TaqI rs731236** Exon 9 c.1056T>C (*T>t*) p.Ile352=	Genotypes (%)	T/T (*T/T*)	31 (94)	14 (82)	0.19
		T/C (*T/t*)	2 (6)	3 (18)	
		C/C (*t/t*)	0 (0)	0 (0)	
	Alleles (%)	T (*T*)	64 (97)	31 (91)	0.20
		C (*t*)	2 (3)	3 (9)	
	HW (p)		1	1	

Tannerella Forsythia								
**Haplotype**				**Total frequency**	**Clinical groups frequency**		**p-value**	**OR (95% CI)**
**FokI**	**BsmI**	**ApaI**	**TaqI**		**LOW**	**HIGH**		
T *(f)*	G *(b)*	C *(a)*	T *(T)*	0.287	0.221	0.465	**0.009**	3.183(1.2977.811)
C *(F)*	G *(b)*	C *(a)*	T *(T)*	0.260	0.309	0.09	**0.02**	0.248(0.071-0.859)
C *(F)*	G *(b)*	A *(A)*	T *(T)*	0.200	0.184	0.283	0.24	1.786 (0.673-4.738)
T *(f)*	G *(b)*	A *(A)*	T *(T)*	0.112	0.133	0.03	0.12	0.237 (0.033-1.699)
C *(F)*	A *(B)*	C *(a)*	T *(T)*	0.05	0.08	0	0.08	-

Prevotella Intermedia					
			LOW (N=27)	HIGH (N=23)	P value
**VDR FokI rs2228570** Exon2 c.2T>C (*f*>*F*)* *p.Met1Thr*	Genotypes (%)	C/C (F/F)	13 (48)	1 (4)	**0.002382**
		C/T (*F/f*)	9 (33)	16 (70)	
		T/T (*f/f*)	5 (19)	6 (26)	
	Alleles (%)	C (*F*)	35 (65)	18 (39)	**0.010352**
		T (*f*)	19 (35)	28 (61)	
	HW (p)		0.2	0.074	
**VDR BsmI rs1544410** Intron 8 c.1024+283A>G(*B*>*b*)*	Genotypes (%)	A/A (*B/B*)	1 (4)	0 (0)	0.066
		A/G (*B/b*)	7 (26)	1 (4)	
		G/G (*b/b*)	19 (70)	22 (96)	
	Alleles (%)	A (*B*)	1 (2)	9 (17)	**0.016089**
		G (*b*)	45 (98)	45 (83)	
	HW (p)		0.55	1	
**VDR ApaI rs7975232** Intron 8 c.1025-49A>C(*A*>*c*)*	Genotypes (%)	A/A (*A/A*)	4 (4)	1 (15)	0.27
		A/C (*A/a*)	15 (48)	11 (56)	
		C/C (*a/a*)	8 (48)	11 (30)	
	Alleles (%)	A (*A*)	23 (43)	13 (36)	0.13
		C (*a*)	31 (57)	33 (64)	
	HW (p)		0.7	0.63	
**VDR TaqI rs731236** Exon 9 c.1056T>C (*T*>*t*) p.Ile352=	Genotypes (%)	T/T (*T/T*)	25 (93)	20 (87)	0.50
		T/C (*T/t*)	2 (7)	3 (13)	
		C/C (*t/t*)	0 (0)	0 (0)	
	Alleles (%)	T (*T*)	52 (96)	43 (93)	0.51
		C (*t*)	2 (4)	3 (7)	
	HW (p)		1	1	

Prevotella Intermedia								
**Haplotype**				**Total frequency**	**Clinical groups frequency**		**p-value**	**OR (95% CI)**
**FokI**	**BsmI**	**ApaI**	**TaqI**		**LOW**	**HIGH**		
T *(f)*	G *(b)*	C *(a)*	T *(T)*	0.287	0.125	0.515	**0.000022**	7.463 (2.809-20.745)
C *(F)*	G *(b)*	C *(a)*	T *(T)*	0.260	0.300	0.144	**0.05**	0.384 (0.139-1.056)
C *(F)*	G *(b)*	A *(A)*	T *(T)*	0.200	0.231	0.224	0.91	0.950 (0.370-2.440)
T *(f)*	G *(b)*	A *(A)*	T *(T)*	0.112	0.157	0.02	**0.02**	0.156 (0.024-1.021)
C *(F)*	A *(B)*	C *(a)*	T *(T)*	0.05	0.09	0.02	0.14	0.203 (0.032-1.283)

Fusobacter Nucleatum					
			LOW (N=21)	HIGH (N=29)	P value
**VDR FokI** **rs2228570** Exon2 c.2T>C (*f*>*F*)* *p.Met1Thr*	Genotypes (%)	C/C (F/F)	5 (24)	9 (31)	0.32
		C/T (*F/f*)	13 (62)	12 (41)	
		T/T (*f/f*)	3 (14)	8 (28)	
	Alleles (%)	C (*F*)	23 (55)	30 (52)	0.76
		T (*f*)	19 (45)	28 (48)	
	HW (p)		0.39	0.46	
**VDR BsmI** **rs1544410** Intron 8 c.1024+283A>G(*B*>*b*)*	Genotypes (%)	A/A (*B/B*)	1 (5)	0 (0)	**0.049991**
		A/G (*B/b*)	6 (29)	2 (7)	
		G/G (*b/b*)	14 (67)	27 (93)	
	Alleles (%)	A (*B*)	8 (19)	2 (3)	**0.010305**
		G (*b*)	34 (81)	56 (97)	
	HW (p)		0.56	1	
**VDR ApaI** **rs7975232** Intron 8 c.1025-49A>C(*A*>*c*)*	Genotypes (%)	A/A (*A/A*)	0 (0)	5 (17)	0.10
		A/C (*A/a*)	11 (52)	15 (52)	
		C/C (*a/a*)	10 (48)	9 (31)	
	Alleles (%)	A (*A*)	11 (26)	25 (43)	0.08
		C (*a*)	31 (74)	33 (57)	
	HW (p)		0.26	1	
**VDR TaqI** **rs731236** Exon 9 c.1056T>C (*T*>*t*) p.Ile352=	Genotypes (%)	T/T (*T/T*)	19 (90)	26 (90)	0.92
		T/C (*T/t*)	2 (10)	3 (10)	
		C/C (*t/t*)	0 (0)	0 (0)	
	Alleles (%)	T (*T*)	40 (95)	55 (95)	0.92
		C (*t*)	2 (5)	3 (5)	
	HW (p)		1	1	

Fusobacter Nucleatum								
Haplotype				Total frequency	Clinical groups frequency		p-value	OR (95% CI)
FokI	BsmI	ApaI	TaqI		LOW	HIGH		
T *(f)*	G *(b)*	C *(a)*	T *(T)*	0.287	0.403	0.275	*0.19*	0.570 (0.245-1.329)
C *(F)*	G *(b)*	C *(a)*	T *(T)*	0.260	0.204	0.275	*0.39*	1.504 (0.583-3.880)
C *(F)*	G *(b)*	A *(A)*	T *(T)*	0.200	0.162	0.206	*0.56*	1.358 (0.481-3.843)
T *(f)*	G *(b)*	A *(A)*	T *(T)*	0.112	0	0.155	***0.0071***	-
C *(F)*	A *(B)*	C *(a)*	T *(T)*	0.05	0.08	0	***0.04***	-
